# Optimization of culture conditions for endophytic bacteria in mangrove plants and isolation and identification of bacteriocin

**DOI:** 10.3389/fphar.2024.1429423

**Published:** 2024-08-02

**Authors:** Jinju Peng, Xingpeng Xie, Tingli Fan, Haotian Ma, Yang Li, Shuaishuai Luo, Mengbo Yu, Yuexia Ding, Yi Ma

**Affiliations:** ^1^ College of Coastal Agricultural Sciences, Guangdong Ocean University, Zhanjiang, China; ^2^ Department of Agricultural and Animal Husbandry Engineering, Cangzhou Technical College, Cangzhou, China

**Keywords:** mangroves, endophytic bacteria, culture, bacteriocin, antibacterial activity

## Abstract

**Introduction:** The antibacterial protein PAG14 was extracted from a metabolite of *Bacillus* G14 isolated from mangrove plants.

**Methods:** In this study, *Pseudomonas aeruginosa*, *Pasteurell multocide*, *Enterobacter aerogenes*, and *Enterococcus faecalis* were used as indicator bacteria to screen endophytes that exhibited antibacterial activity. The endophyte culture conditions were optimized to enhance productivity. Subsequently, the culture supernatant was salted using ammonium sulfate, followed by purification using dextran gel chromatography and ion exchange column techniques. Finally, the structures of antibacterial proteins were identified using mass spectrometry.

**Results and Discussion:** The optimal culture conditions for *Bacillus* G14 were 2% mannitol, 0.5% fish peptone, 0.05% KH2PO4 + 0.05% K2HPO4 + 0.025% MnSO4·H2O. The antibacterial substances exhibited stability within the temperature range of 30–40°C and pH range of 5.0–7.0, while displaying sensitivity toward enzymes. The antibacterial activity decreased as the duration of UV irradiation increased. The antibacterial protein PAG14, isolated from the culture broth of *Bacillus* G14 through purification using dextran gel and ion-exchange columns, was identified as a class III bacteriocin using LC-MS/MS, similar to Lysozyme C. These findings serve as a theoretical foundation for the investigation and application of bacteriocins in food products.

## Introduction

Endophytic bacteria mainly refer to endophytic bacteria in plant tissues, organs, and intercellular spaces. The metabolic pathways unique to plant endophytes has been demonstrated to facilitate the production of a plethora of bioactive substances with novel structures and potent antibacterial effects, thereby serving as a valuable resource for the development of novel antibacterial drugs (Jouda et al., 2016). *Bacillus* species are widely distributed and can be found in various sources, including soil, water, air, and animal intestines, showing diversity and universality (Gond et al., 2015). Senadeera et al. (2012) first proposed the method of “OSMAC,” by changing the culture conditions biologically or abiotically to activate silencing biosynthetic gene clusters to produce new metabolites, secondary metabolites of more structural types were obtained. Bacteriocins are a class of antimicrobial proteins or polypeptides synthesized by bacterial ribosomes (Heilbronner et al., 2021). The secretion of bacteriocins varies among different *Bacillus* strains, exhibiting diversity not only in terms of molecular weight, but also in morphological structure and physical and chemical properties. *Bacillus* bacteriocins are another major class of bacteriocins, following *Lactobacillus* bacteriocins (Abriouel et al., 2011), and numerous bacteriocins have been discovered to date (Mercado and Olmos, 2022). For instance, *Bacillus cereus* can secrete various types of bacteria, such as cerein 7, cerein 7 B, and cerein MRX1 (Oscáriz and Pisabarro, 2000; Bizani and Brandelli, 2002; Oscáriz et al., 2006; Sebei et al., 2007). The literature shows that the isolation and culture conditions of bacteriocin produced by *Bacillus atrophicum* and *Bacillus amyloliquefaciens* were optimized, which effectively increased the production of bacteriocin, and further expanded the understanding of the potential applicability of bacteriocin (Elazzazy et al., 2024). *Bacillus* bacteriocins exhibit non-toxic and innocuous properties and are highly effective in minimizing the likelihood of drug resistance development (Kumariya et al., 2019). Bacteriocins exhibit a broad antibacterial spectrum, exceptional thermal stability, and resistance under both acidic and alkaline conditions (Cotter et al., 2013; Özel et al., 2018).

**FIGURE 1:**
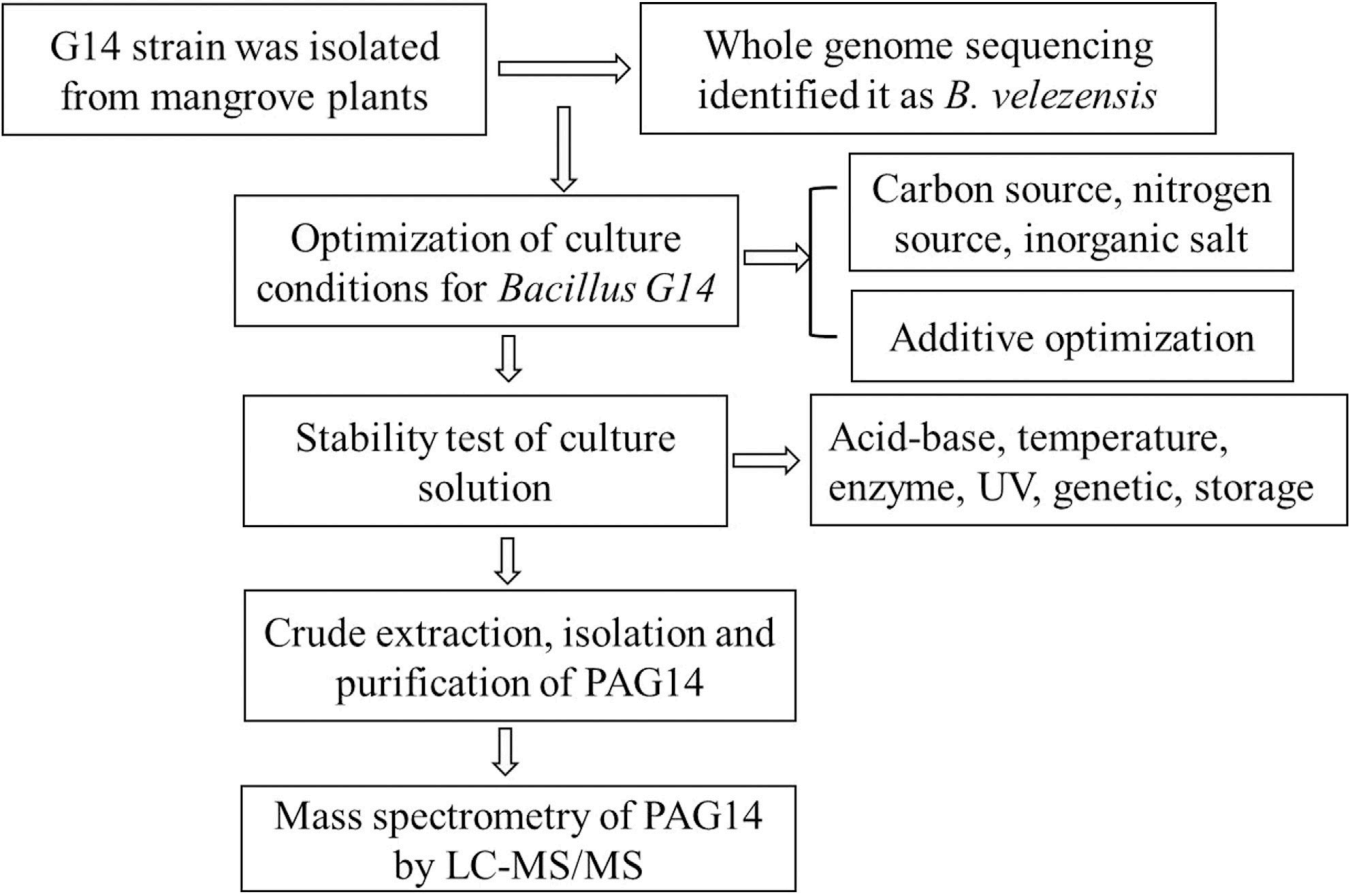
The experimental scheme of this study.

At present, many different bacteriocins have been isolated from *Bacillus* sp., which have been reviewed (Basi-Chipalu et al., 2022; Vaca et al., 2022); however, there are deficiencies in their structure and stability of *Bacillus* bacteriocins. The stability study of *Bacillus* bacteriocins provided strong support for structural studies of *Bacillus* bacteriocins. In this study, strain G14 with a broad-spectrum antibacterial effect was isolated from mangrove plants and identified as *Bacillus velezensis* by whole gene sequencing analysis. The study had shown that the bactericidal activity of *B. velezensis* bacteriocin is wider than the other bacteriocins (Vaca et al., 2023). The culture conditions of the strain were optimized to determine the stability of active antibacterial substances. *Bacillus* G14 culture supernatant was purified using dextran gel chromatography and ion-exchange columns. The structure of the purified antibacterial protein was identified using mass spectrometry. The experimental scheme of this study was shown in Figure 1. It provides valuable theoretical references for subsequent applications (such as food, health supplements, animal husbandry, feed production, and aquaculture) as well as research and development.

## Materials and methods

### Reagents and strains

To improve bacteriocin production, the following substances were selected for medium optimization according to a preliminary experiment. Glucose, mannitol, soluble starch, lactose, fructose, and sucrose were purchased from Shanghai Maclin Biotechnology Co., Ltd., China. Beef paste, peptone, casein peptone, fish peptone, beef powder, and tryptone were purchased from Beijing Aoboxing Biotechnology Co., Ltd., China. NaCl, MnSO_4_, CaCl_2_, KCl, MnSO_4_·H_2_O, NaH_2_PO_4_, Na_2_PO_4_, KH_2_PO_4_, K_2_HPO_4_ were produced by Shanghai Yien Chemical Technology Co., LTD., China.

*Pseudomonas aeruginosa* (CVCC393), *Enterobacter aerogenes* (ATCC13048), *Enterococcus faecalis* (ATCC29212), and *Pasteurea multocida* (ATCC9027) were used as indicator bacteria and purchased from Beijing Reserve Biotechnology Co., Ltd., China.

### Optimization of single component of culture medium

Bacteria were isolated from the tissues of mangrove plants, and endophyte G14 was screened for its antibacterial activity against *P. aeruginosa*, *Pasteurella multocida*, *E. aerogenes* and *E. faecalis*, was selected and identified as *B. velezensis. Bacillus* G14 was inoculated into LB medium and cultured overnight at 30°C, 180 r/min. It was added to various culture media at a 2% concentration and incubated for 24 h under the same conditions. Antibacterial effects were detected using the agar plate method. Indicator bacteria were introduced into the LB solid medium, and a sterile perforator was used. The supernatant of the culture medium was filtered by 0.22 μm filter membrane and added into the agar plate. The diameter of the inhibition zone was measured after culture at 28°C for 24 h.

The carbon source medium was composed of 10.0 g/L peptone, 3.0 g/L beef powder, and 5.0 g/L NaCl, while the basic medium was supplemented with 4% mannitol, lactose, fructose, sucrose, glucose, and soluble starch respectively.

The optimal carbon source, mannitol, was added to the nitrogen-source medium. The basal medium for the nitrogen source consisted of 40.0 g/L mannitol and 5.0 g/L NaCl, while the basic medium was enriched with 2% beef extract, peptone, casein peptone, fish peptone, beef powder, and tryptone individually.

The composition of the inorganic salt base medium was as follows: 40.0 g/L mannitol, 20.0 g/L fish peptone, and the basal medium was supplemented with 0.5% NaCl, 0.5% MnSO_4_, 0.5% CaCl_2_, 0.5% KCl, 0.5% MnSO_4_·H_2_O, 0.5% NaH_2_PO_4,_ 0.5% Na_2_PO_4_, 0.5% KH_2_PO_4_, 0.5% K_2_HPO_4_, 0.25% NaH_2_PO_4_ + 0.25% Na_2_HPO, 0.2% KH_2_PO_4_ + 0.2% K_2_HPO_4_ + 0.1% MnSO_4_·H2O, 0.2% NaH_2_PO_4_ + 0.2% Na_2_HPO_4_ + 0.1% MnSO_4_·H_2_O, respectively.

### Optimization of carbon source, nitrogen source and inorganic salt content in culture medium

The optimal carbon and nitrogen sources and inorganic salt were selected as the basic media. To optimize the amount of the carbon source additive, mannitol was added at mass volume fractions of 1%, 2%, 4%, 8%, and 16%. The optimal amount of fish peptone, a nitrogen source additive, was determined based on the concentrations of 0.5%, 1%, 2%, 4%, and 8%. The optimal amount of inorganic salt additive, consisting of KH_2_PO_4_ + K_2_HPO_4_ + MnSO_4_·H_2_O, was added based on the percentages of 0.1%, 0.2%, 0.5%, 1% and 2% for further optimization. The antibacterial activities of the culture supernatants were determined under identical experimental conditions. All experiments were conducted in triplicates.

The optimal amounts of carbon, nitrogen, and inorganic salts were determined based on the results of a single-factor experiment. Subsequently, an orthogonal experimental design was used to perform a three-factor, three-level analysis to select the optimal quantities of carbon, nitrogen, and inorganic salt additives. All experiments were conducted in triplicates.

### Stability test of culture solution

#### Acid-base stability

The supernatant of the culture liquid was adjusted to pH values of 8.0, 9.0, 10.0, 11.0, 12.0, 13.0 and 14.0 using a solution of NaOH with a concentration of 1 mol/L, and to pH values of 1.0, 2.0, 3.0, 4.0, 5.0 and 6.0 using a solution of HCl with a concentration of 1 mol/L respectively. After incubation at a temperature of 4°C for a duration of 12 h, the pH was restored back to neutral, the antibacterial activity was detected.

#### Temperature stability

The supernatant of the culture liquid was subjected to thermal treatments at 40°C, 60°C, 80°C, and 100°C for a duration of 30 min each, followed by a sterilization process at 121°C for an additional 30 min to detect the antibacterial activity.

#### Enzyme stability

The culture supernatant was treated with 1 mg/mL pepsin, trypsin, papain, or protease K. Subsequently, the antibacterial activity was detected following incubation in a water bath at 37°C for 1 h.

#### UV stability

The supernatant of the culture liquid was exposed to a 15 W UV lamp at a distance of approximately 15 cm for 1, 2, 3, 4 and 5 h to detect antibacterial activity.

#### Genetic stability

*Bacillus* G14 preserved at −80°C was inoculated into LB liquid medium and cultured at 30°C for 24 h as the first generation. The second generation was obtained by marking and culturing the LB solid culture at 30°C for 24 h. A single colony from the second generation was inoculated into the LB liquid medium to obtain a third generation. Following this method, the culture was transferred to the 11th generation. The 1st, 3rd, 5th, 7th, 9th, and 11th generations of bacteria were fermented and cultured to determine their antibacterial activities.

#### Storage stability

The supernatant of the culture liquid was stored at 4°C and −20°C, and 5 time points were set for each storage condition, which were 0, 3, 6, 9, and 12 days, respectively. The next step was to detect the antimicrobial activity over time. Samples were collected at different time points for analysis.

These tests were conducted in triplicate using the untreated culture broth supernatant as the positive control.

### Crude extraction of antibacterial protein

The optimized culture conditions for culturing *Bacillus* G14 included a liquid volume of 30 mL, an inoculation volume of 2%, a culture temperature of 30°C, and a culture time of 120 h. The culture solution was centrifuged at 4°C at 8,000 × g for 30 min and subsequently filtered using a 0.22 μm filter membrane. The sterile supernatant was packed into a 1 KDa dialysis bag and concentrated 10 times. The antibacterial activity of the concentrated culture supernatant was verified using the solid AGAR drilling method with *Streptococcus* as the indicator bacteria.

The supernatant was salted out with 50%, 60%, 70%, and 80% saturation ammonium sulfate respectively, centrifuged at 4°C at 8,000 × g for 30 min, and the precipitated proteins were collected. Ammonium sulfate was removed by dialysis. The retained molecular weight of the dialysis bag was 1 kDa, and the bag was placed in PBS (a phosphate-buffered saline (PBS) solution (pH 5.5 for dialysis. After dialysis, the antibacterial protein was filtered by 0.22 μm filter membrane. The solid AGAR drilling method was used to verify the antibacterial activity of the protein, and the optimal concentration of ammonium sulfate salting was determined based on different antibacterial effects.

### Purification of antibacterial protein using glycan gel chromatography column

The antibacterial proteins were purified using ACHROM Firin Plus [Taidu Biotechnology (Suzhou) Co., Ltd., China]. Using a Tideroe-GF75 gel chromatographic column with deionized water as the mobile phase equilibrium chromatography column. The crude protein extraction solution was sampled at a flow rate of 0.55 mL/min and detected at 280 nm, followed by the collection of protein isolates. The antibacterial activities of the different isolated proteins were verified using the solid AGAR drilling method.

### Purification of protein using ion exchange column

A Titrap Q HP ion-exchange column was used to isolate and purify the antibacterial proteins. The purified column was equilibrated with 0.05 mol/L of sterile NaCl, and samples were collected at a flow rate of 3 mL/min for detection at 280 nm. The antibacterial activities of the different isolated proteins were verified using the solid AGAR drilling method.

### Mass spectrometry of antibacterial protein PAG14

Purified antibacterial protein PAG14 powder, which underwent vacuum freeze-drying, was sent to Beijing Beitapak Biotechnology Co., Ltd.

The samples were reduced and alkylated, followed by their addition to a dithiothreitol (DTT) solution to achieve a final concentration of 10 mmol/L. The samples underwent reduction in a water bath at 56°C for 1 h. The IAA (Iodoacetamide, IAA) solution was prepared at a final concentration of 50 mmol/L, and the reaction was allowed to proceed in the dark for 40 min. The samples were desalted using a self-filling column prior to LC-MS/MS analysis (Thermo Fisher Scientific). For the identification of polypeptide fragments. The primary mass spectrometry scan range spanned from 350 to 1800 (m/z) with a resolution of 70,000, whereas the secondary MS/MS resolution was set at 17,500.

Amino acid sequences of PAG14 were obtained using MaxQuant software (Max-Planck-Institute of Biochemistry, Germany) and the Universal Protein Resource (UniProt; https://www.uniprot. org/blast/) and the Antimicrobial Peptide Database (APD; https://aps. unmc.edu/AP/) were used to compare the sequences with known AMP amino acid sequences of antimicrobial peptides (Zou et al., 2018).

## Results

### Single component screening of culture medium solution

The results of the single-component screening of the culture medium are presented in Table 1. Mannitol as a single carbon source, fish peptone as a single nitrogen source and KH_2_PO_4_+K_2_HPO_4_+MnSO_4_·H_2_O as a single inorganic salt, they were effective against all four indicator bacteria and had the largest inhibition zone diameter, which had the best influence on inhibitory effect, respectively.

**TABLE 1:** Inhibition effect of carbon source, nitrogen source and inorganic salt on culture solution.

Medium	Ingredient	Inhibition zone diameter (mm)			
		*Pseudomonas aeruginosa*	*Enterobacter aerogenes*	*Pasteurella multocida*	*Enterococcus faecalis*
Carbon source	Control		11.05	15.87	
	Glucose		12.12	16.14	15.21
	Mannitol	10.25	17.34	17.41	17.21
	Soluble starch		15.34	16.31	13.12
	Lactose	14.17	10.31	17.05	15.04
	Levulose		11.16	18.12	13.04
	Saccharose		9.16	11.18	12.14
Nitrogen source	Beef extract			9.07	
	Peptone			11.23	
	Casein peptone	9.08	10.56	11.12	11.89
	Fish peptone	9.15	10.14	14.15	12.16
	Beef powder			13.87	12.02
	Tryptone		12.23	12.08	13.14
	Beef extract			9.14	
Inorganic salt	No inorganic salt control				
	NaCl	9.07		14.87	12.03
	K_2_HPO_4_		9.17	15.34	11.23
	MnSO_4_	11.16	10.13	16.12	
	CaCl_2_	9.89	12.11	11.12	10.14
	KCl			10.04	
	MnSO_4_·H_2_O	12.13	12.34	14.25	14.82
	NaH_2_PO_4_ + Na_2_HPO_4_			13.87	11.12
	NaH_2_PO_4_				10.42
	Na_2_HPO_4_			11.67	11.32
	KH_2_PO_4_			10.23	10.94
	KH_2_PO_4_ + K_2_HPO_4_ + MnSO_4_·H_2_O	14.21	13.06	20.78	15.47
	NaH_2_PO_4_ + Na_2_HPO_4_ + MnSO_4_·H_2_0	11.94	11.97	17.16	15.06

### Optimization of each element added amount

As shown in Table 2, the additive amounts of mannitol were 2%, 4%, and 8%, and the culture solution had an inhibitory effect on the four indicator bacteria. The additive amounts of fish peptone were 0.5%, 1%, and 2%; the culture solution had an inhibitory effect on all four indicator bacteria, and the inhibition zone diameter was larger. The added amounts of inorganic salts were 0.125%, 0.25%, and 0.5%, and the culture solution exhibited inhibitory effects on all four indicator bacteria, with larger inhibition zone diameters indicating stronger inhibitory effects. In summary, three additive amounts of mannitol, fish peptone, and inorganic salt were selected for the subsequent orthogonal experiments.

**TABLE 2:** Inhibition effect of mannitol, fish peptone and inorganic salt addition on culture solution.

	Additive ratio (%)	Inhibition zone diameter (mm)			
		*Pseudomonas aeruginosa*	*Enterobacter aerogenes*	*Pasteurella multocida*	*Enterococcus faecalis*
Additive amount of mannitol	1	12.66 ± 0.61	—	—	—
	2	11.75 ± 0.53	9.46 ± 0.49	13.48 ± 1.22	14.13 ± 1.83
	4	11.47 ± 0.59	9.56 ± 0.53	11.89 ± 0.64	14.75 ± 0.56
	8	10.71 ± 0.56	10.22 ± 0.97	12.39 ± 0.45	13.38 ± 0.21
	16	—	—	—	13.09 ± 0.13
Additive amount of fish peptone	0.5	9.66 ± 1.05	11.84 ± 0.61	16.86 ± 0.56	16.47 ± 1.45
	1	9.33 ± 0.58	11.49 ± 0.57	17.92 ± 0.49	17.46 ± 0.59
	2	9.44 ± 0.30	10.63 ± 0.68	19.53 ± 0.62	13.83 ± 0.50
	4	11.98 ± 1.15	9.47 ± 0.64	10.48 ± 0.57	13.53 ± 0.46
	8	9.61 ± 0.73	9.32 ± 0.45	—	9.50 ± 0.67
Additive amount of inorganic salt	0.125	11.23 ± 1.11	9.47 ± 0.64	12.31 ± 0.83	14.49 ± 0.49
	0.25	10.44 ± 0.76	9.72 ± 0.53	13.85 ± 0.52	14.61 ± 0.54
	0.5	9.31 ± 0.54	10.03 ± 0.89	17.79 ± 1.17	14.96 ± 0.72
	1	9.84 ± 0.58	11.83 ± 0.66	11.69 ± 0.65	13.81 ± 0.68
	2	9.48 ± 0.64	9.51 ± 0.58	9.69 ± 0.58	—

### Optimization of culture conditions by orthogonal experiment

The orthogonal experiment of L9 (34) was designed by using SPSS software with mannitol, fish peptone, KH_2_PO_4_ + K_2_HPO_4_ + MnSO_4_·H_2_O as test factors. The design scheme is presented in Table 3.

**TABLE 3:** Orthogonal experimental design.

Experimental group	Carbon source (%)	Nitrogen source (%)	Inorganic salt (%)
1	2	0.50	0.125
2	2	1	0.25
3	2	2	0.50
4	4	0.50	0.25
5	4	1	0.50
6	4	2	0.125
7	8	0.50	0.50
8	8	1	0.125
9	8	2	0.25

As shown in Table 4, among experimental groups 1–9 designed by the orthogonal experiment, group 1 exhibited the largest antibacterial circle diameter and the most effective antibacterial effect. Therefore, the optimal culture conditions for strain G14 were determined to be 2% mannitol, 0.5% fish peptone, and 0.05% KH_2_PO_4_ + 0.05% K_2_HPO_4_ + 0.025% MnSO_4_·H_2_O.

**TABLE 4:** Inhibition effect of different fermentation broth in orthogonal experiment.

	Inhibition zone diameter (mm)			
Experimental group	*Pseudomonas aeruginosa*	*Enterobacter aerogenes*	*Pasteurella multocida*	*Enterococcus faecalis*
1	11.38 ± 1.09	11.49 ± 0.96	19.72 ± 0.68	22.62 ± 0.84
2	10.08 ± 0.98	10.94 ± 1.19	19.67 ± 0.42	19.49 ± 0.48
3	9.22 ± 0.55	10.69 ± 0.43	12.44 ± 0.59	13.98 ± 0.75
4	12.38 ± 0.33	9.46 ± 0.51	19.19 ± 0.41	18.51 ± 0.64
5	9.95 ± 0.67	10.68 ± 0.51	19.38 ± 0.42	19.35 ± 0.47
6	10.34 ± 0.63	—	17.49 ± 0.83	14.88 ± 0.37
7	11.24 ± 0.77	—	17.11 ± 0.39	17.54 ± 0.31
8	9.55 ± 0.69	—	16.64 ± 0.49	12.47 ± 0.51
9	9.54 ± 0.48	—	11.44 ± 1.14	13.62 ± 0.51

### Stability test of culture solution

**FIGURE 2:**
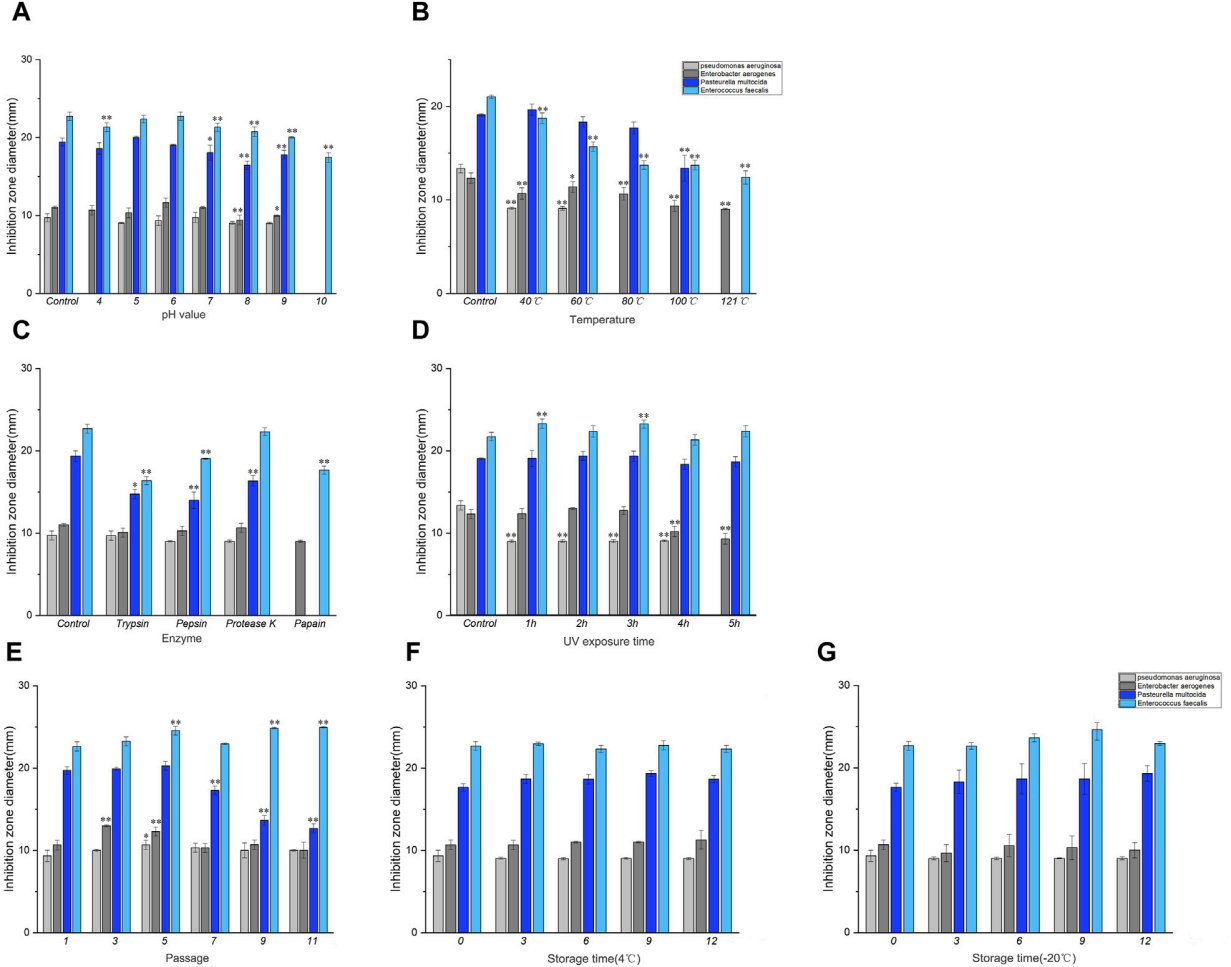
Results of stability determination of culture solution. **(A)** Acid-base stability, **(B)** Temperature stability, **(C)** Enzyme stability, **(D)** UV stability. Note: * indicates significant difference (*P < 0.05*), ** indicates highly significant difference (*P < 0.01*). Results of stability determination of culture solution (continued). **(E)** Genetic stability, **(F)** 4°C storage stability, **(G)** −20°C storage stability. Where Control is the control check.

As shown in Figure 2A, the pH of the control group corresponded to the pH of the culture solution (5.5) during bacterial isolation and culture. Different pH values exhibited significant variations in their impact on the antibacterial activity compared to the control group. When the pH of the culture medium was below 5.0 or above 7.0, there was a gradual decline in the antibacterial efficacy with changing pH levels. However, at pH 5.5, the culture solution exhibited the highest antibacterial activity.

As shown in Figures 2B, the temperature of the control group was 30°C when the bacteria were isolated and cultured. Compared with the control group, the inhibitory effect of the culture liquid decreased at 40–60°C after being treated at different temperatures, and the antibacterial activity of the culture liquid against *P. aeruginosa*, *E. aerogenes* and *E. faecalis* was significantly different compared with the control group (*P < 0.01*). No significant differences were observed in *P. multocida*. At temperatures ranging from 80°C to 121°C, there was a complete loss of antibacterial activity against *P. aeruginosa*. Additionally, a substantial decrease in antibacterial activity against *E. aerogenes* and *E. faecalis* was observed (*P < 0.01*), and the antibacterial activity against *P. multocida* disappeared at 121°C. The results showed that the culture solution was stable at 30–60°C.

As shown in Figure 2C, the control group was not subjected to enzyme treatment. Compared to the control group, the antibacterial activity of the culture broth treated with trypsin and pepsin against *P. multocida* and *E. faecalis* decreased. The antibacterial activity of the culture broth treated with protease K showed a decline against *P. multocida*. Furthermore, papain treatment decreases the antibacterial activity of the culture broth against *E. aerogenes* and *E. faecalis*. However, it completely disappeared for *P. aeruginosa* and *P. multocida*. Henceforth, it can be concluded that papain displayed higher sensitivity towards antimicrobial protein.

Compared to the control group, the antibacterial activity of the culture solution decreased within 4 h under UV light after different durations of UV treatment, as depicted in Figure 2D. The antibacterial activity of the culture solution against *P. aeruginosa* was significantly different (*P < 0.01*), and the antibacterial activity of the culture solution against *P. aeruginosa* completely disappeared at 5–6 h. Simultaneously, a significant decrease was noted in the antibacterial activity against *E. aerogenes* (*P < 0.01*).

As depicted in Figure 2E, the antibacterial activity of the culture solution against *P. multocida* gradually decreased from the 5th generation onward when compared to that of the control group (1st generation). However, no significant antibacterial activity was observed against the other indicator strains after passaging.

As depicted in Figures 2F, G, the antibacterial activity of the culture liquid stored at 4°C and −20°C for different durations, remained relatively stable compared to the control group (day 0).

### Isolation and purification of antibacterial protein PAG14

**FIGURE 3:**
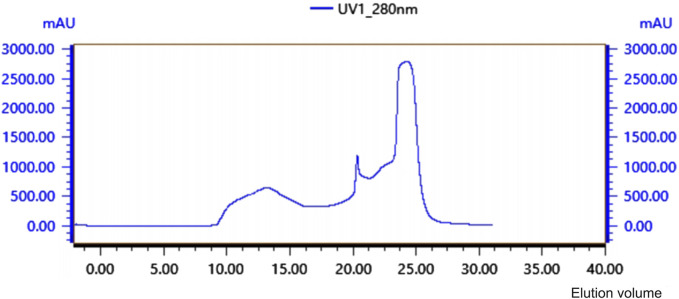
Glycan Gel Chromatographyof antibacterial protein PAG14.

**FIGURE 4:**
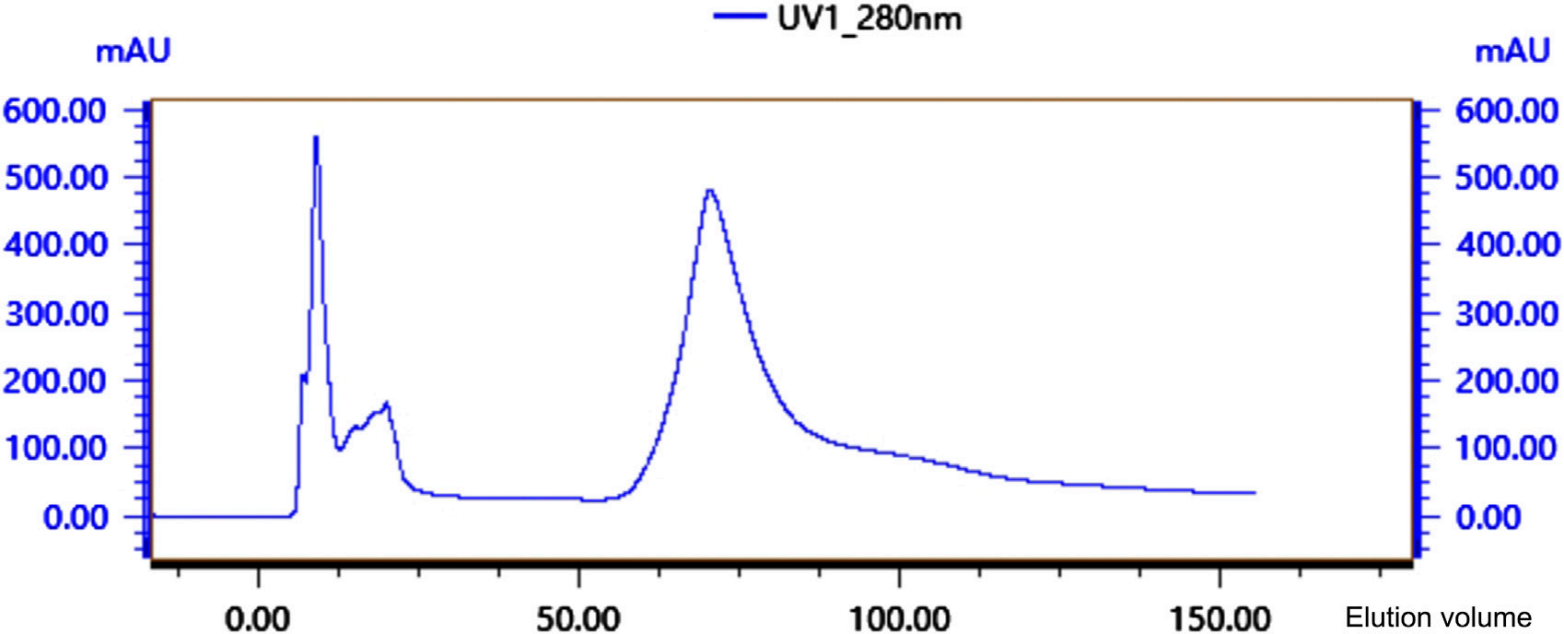
Ion-exchange column chromatography of antibacterial protein PAG14.

**FIGURE 5:**
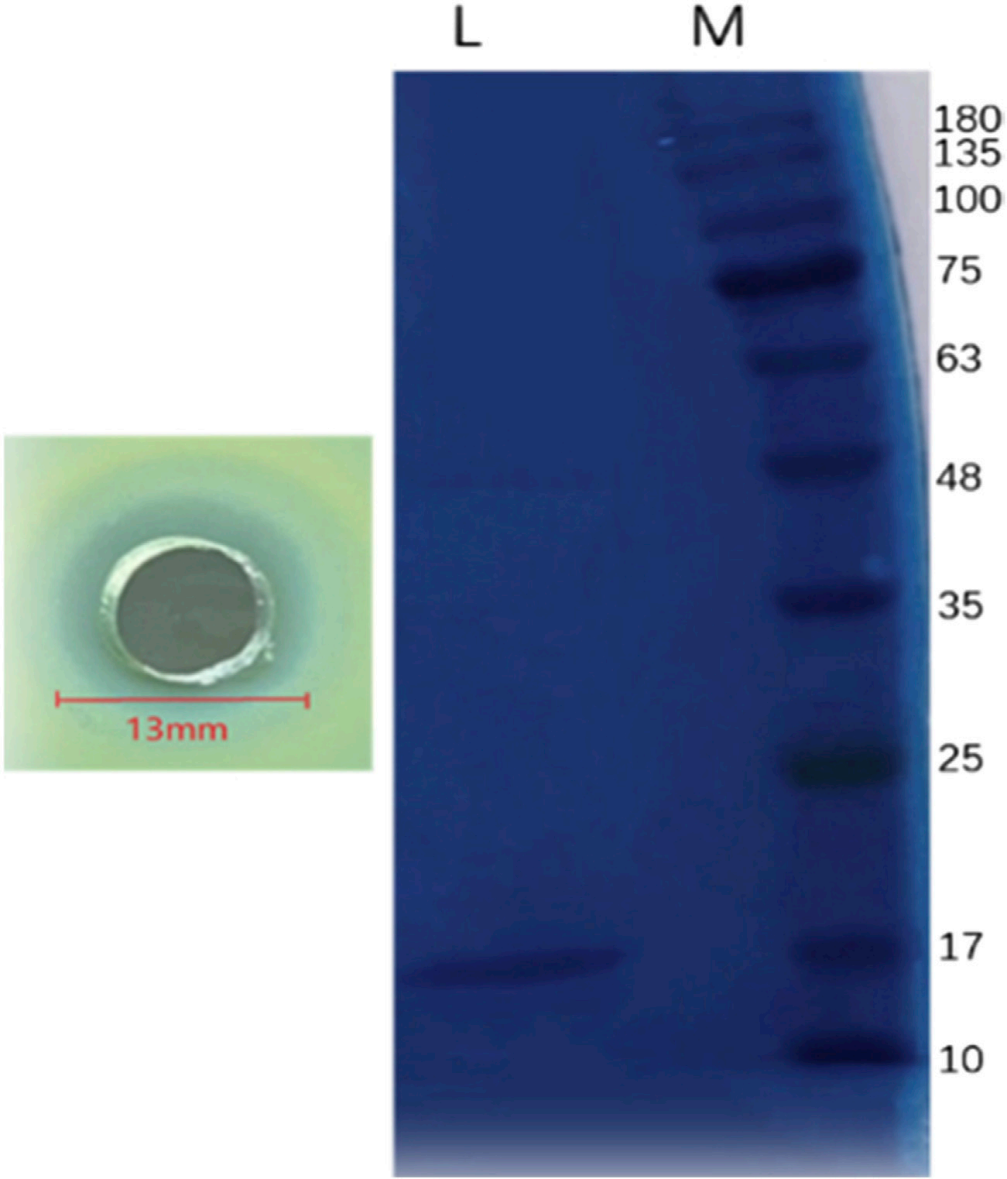
Gel electrophoresis of antibacterial protein PAG14. M: Non-pre-stained protein Marker (10–200 kDa); L: antibacterial protein PAG14.

Crude protein was obtained by salting, resolubilizing, and dialyzing the *Bacillus* G14 culture supernatant. After purification by dextran gel electrophoresis, the results showed that peak 3 protein exhibited antibacterial activity (Figure 3). The results for peak protein 3 after ion purification are shown in Figure 4. Peak 3 protein showed potent antibacterial activity against *P. aeruginosa* with an inhibition zone diameter of 13 mm (Figure 5). SDS-PAGE revealed a purified protein strip with a molecular mass ranging from approximately 11–17 KDa (Figure 4), indicating that the extracted antibacterial protein, PAG14, had a molecular weight within this range.

### Results of LC-MS/MS

The mass spectrum structure of the antibacterial protein PAG14 was analyzed using the Byonic software, as presented in Table 5. Based on the LC-MS/MS results, the identified proteins were comprehensively evaluated considering the score value, sequence coverage rate, and relative molecular mass. Consequently, it was presumed that the antibacterial substance belonged to the class III bacteriocin Lysozyme C. It has been reported in literature that bacteriocin Lysozyme C had a molecular mass of approximately 16.5 KDa (Ling et al., 2024), which further supports the findings obtained from SDS-PAGE analysis.

**TABLE 5:** Results of partial protein identification of LC-MS/MS.

Protein name	Score	Sequence coverage%	Molecular weight/Da
Bifunctional polymyxin resistance protein ArnA	266.9	25	74,186
Lysozyme C	95.46	26	16,537
Immunoglobulin kappa variable	77.03	11	13,143
Immunoglobulin heavy constant alpha alpha 2	62.73	5	42,334
Immunoglobulin heavy constant alpha 1	62.73	5	42,849
Immunoglobulin alpha-2 heavy chain	62.73	2	48,934
Lysosome-associated membrane glycoprotein 1	60.67	3	43,865

## Discussion

The efficiency of bacteriocin production through bacterial strain culture is related to the performance of the strain itself, but is also influenced by various factors, such as culture conditions and processes. The bacteria initially entered the logarithmic growth phase, with exponential growth in bacterial numbers and production of secondary metabolites. The strain reached a stable phase after 18 h and maximum production at approximately 22 h (Liu et al., 2021; Kim et al., 2022). Subsequently, as strain growth plateaued, bacteriocin production ceased. Culture media play a crucial role in bacteriocin production, because nutrients directly affect the expression and synthesis of bacteriocins and other secondary metabolites. The production of secondary metabolites by *Bacillus* is mainly affected by the nutrient composition of the medium (carbon, nitrogen source, and inorganic salt), culture conditions (temperature, pH, culture time, and inoculation amount), and highly complex metabolic regulation mechanisms. The production of inhibitory substances by microorganisms varies under different culture conditions. In this study, a single-factor orthogonal experiment was conducted to investigate the impact of optimal carbon and nitrogen sources, inorganic salts, and their ratios on the antibacterial activity of *Bacillus* G14 culture liquid. The optimum conditions were 2% mannitol, 0.5% fish peptone, 0.05% KH_2_PO_4_ + 0.05% K_2_HPO_4_ + 0.025% MnSO_4_·H_2_O, and the culture solution exhibited the best inhibitory effect. A study conducted by Anthony et al. revealed that elevated levels of yeast extract and NaCl, along with alkaline pH, high temperature, and vigorous agitation, significantly enhanced bacteriocin production in B. licheniformis AnBa9 (Anthony et al., 2009). Media and culture conditions must be carefully chosen to maximize bacteriocin production in *Bacillus* species.The selection of nutrients and the acquisition of appropriate concentration in the single factor culture medium provided a direct basis for the production of antibacterial substances by *Bacillus* G14.

The stability experiment of *Bacillus* G14 demonstrated that the antibacterial substance remained stable within a temperature range of 30–40°C during culture, whereas its activity significantly declined when exposed to temperatures exceeding 80°C. Hong et al. found that the activity of Bacteriocin from the *B. subtilis* was 100% after incubation at 0–50°C for 12 h but decreased to 70% after incubation at 70°C for 12 h (Hong et al., 2022). In contrast, the antimicrobial activity of a substance produced by *Bacillus subtilis* against *B. cereus* was lost after incubation at 80°C or 100°C for 1 h, and the activity against *Listeria monocytogenes* decreased by 50% after incubation at 60–80°C for 15 min (Kamoun et al., 2005; Kindoli et al., 2012). The culture material of *Bacillus* G14 was decomposed by UV irradiation, resulting in decreased antibacterial activity with increasing irradiation time. The pH stability test of the antibacterial substance from *Bacillus* G14 revealed that its activity was higher within a pH range of 5.0–7.0 in the culture solution, suggesting that acidic conditions could enhance the yield of the antibacterial substance. The stability of bacteriocins limits their application. For example, lactostreptococcin (Nisin) has strong activity in acidic environments, but loses activity in neutral and alkaline environments (Haider et al., 2022). It has been previously reported that the activity of the antimicrobial substance produced by *B. subtilis* SC-8 is lower at pH 3 than at pH 4–10. Bacthruricin F4, produced by *Bacillus thuringiensis* showed 40% residual antimicrobial activity at pH 8 and approximately 80% at pH 3 (Lee et al., 2010). The bacteriocin isolated from *Bacillus* BS2 was stable at pH 4–9 (Lee et al., 2022). The antibacterial substance of *Bacillus* G14 was sensitive to trypsin, pepsin, papain, and protease K. The antimicrobial activity of bacteriocins is lost upon exposure to proteolytic enzymes such as protease, proteinase K, and pronase E, confirming that the purified substance is proteinaceous (Chen et al., 2018). The antibacterial activity of *Bacillus* G14 against *P. multocida* decreased significantly in 7–11 generations. These excellent properties make *Bacillus* G14 bacteriocins have potential application prospects in feed processing and drug therapy.

Bacteriocins are ribosomally synthesized peptides that exert antibacterial effects against strains of the same species, or species that are more distantly related to the bacteriocin-producer (Cleveland et al., 2001). Bacteriocins have great potential in pharmaceutical, agricultural, and biochemical engineering industries (Iqbal et al., 2016). Rapid advancements in protein separation and purification technologies have led to the increasing recognition and application of a greater number of active substances derived from *Bacillus*. Wei et al. isolated subtilin JS-4 from *B. subtilis*, which inhibited the growth of *Listeria* (Wei et al., 2021). Kim et al. (2024) isolated *Bacillus* bacteriocins from fermented food and found that they inhibited the growth of aerobic bacteria and fungi on cheese surfaces. Pinontoan et al. (2018) isolated *B. subtilis* G8 with fibrinolytic activity from natto. Watanakij et al. (2020) isolated the extracellular fraction of *B. subtilis* BCC 42005 isolated from African locust bean potentially possessed aflatoxin B-1-degrading ability. Bacteriocins increase the permeability of the cell membrane, cause electrolyte outflow from the cell, interfere with proton dynamics, and cause ATP leakage (Willey and Van, 2007). According to their molecular structure characteristics of bacteriocins, *Bacillus* bacteriocins can be divided into three classes (Zgheib et al., 2020; Farid et al., 2023): Class I, modified bacteriocins, also known as lanthiobacitins, small molecule polypeptides (<5 ku); Class II, unmodified bacteriocins, and linear small molecule polypeptides (0.77–10 ku). Class III is a heat-labile protein-like bacteriocin (>10 ku), which is an unmodified protein of high molecular mass, including antibacterial proteins that cannot be classified owing to incomplete genetic, molecular, or amino acid structure data (i.e., bacteriocins). The mechanism of action of these bacteriocins involves inducing gram-positive bacterial cell wall alterations and gram-negative bacterial outer membrane disorganization, changing their structures, and causing cell lysis (Abriouel et al., 2011). Vaca et al. (2022) showed that most of the antibacterial substances synthesized by *Bacillus* were bacteriocins or bacteriocin-like substances. In this study, the antibacterial protein PAG14, isolated from the metabolite of *Bacillus* G14, was classified as a class III bacteriocin by LC-MS/MS, similar to Lysozyme C. Studies had shown that *B. velezensis* and *B. subtilis* encode several related secondary metabolite gene clusters, which can effectively inhibit the growth activity of Gram-negative bacteria (Xiang et al., 2021; Soni et al., 2022). We found that the secondary metabolites of *B. velezensis* G14 can inhibit a variety of pathogens which has broad application prospect in the future development.

## Conclusion

The optimal culture conditions of G14 were determined to be 2% mannitol, 0.5% fish peptone, 0.05% KH_2_PO_4_ + 0.05% K_2_HPO_4_ + 0.025% MnSO_4_·H_2_O, with the antibacterial substances exhibiting stability at temperatures ranging from 30 to 40°C. The activity of these substances decreased with increasing UV irradiation time, whereas their effectiveness was highest when the pH value of the culture solution ranged between 5.0 and 7.0. Additionally, it was discovered that the antimicrobial substances were sensitive to trypsin, pepsin, papain and protease K enzymes. Furthermore, a class III bacteriocin produced by *Bacillus* G14 was purified and identified as being responsible for producing the antibacterial protein PAG14, which exhibits strong inhibitory effects on *P. aeruginosa*, with a molecular mass of approximately 11–17 kDa.

## Data Availability

The original contributions presented in the study are included in the article/supplementary material, further inquiries can be directed to the corresponding authors.
